# Evaluation of Specimen Types for Pima CD4 Point-of-Care Testing: Advantages of Fingerstick Blood Collection into an EDTA Microtube

**DOI:** 10.1371/journal.pone.0202018

**Published:** 2018-08-23

**Authors:** Luciana Kohatsu, Omotayo Bolu, Mary E. Schmitz, Karen Chang, Ruth Lemwayi, Nichole Arnett, Michael Mwasekaga, John Nkengasong, Fausta Mosha, Larry E. Westerman

**Affiliations:** 1 United States Centers for Disease Control & Prevention, Center for Global Health, Division of Global HIV/AIDS, Atlanta, Georgia, United States of America; 2 United States Centers for Disease Control & Prevention, Dar es Salaam, Tanzania; 3 Allan Rosenfield Global Health Fellowship, Association of Schools and Programs of Public Health, Washington DC, United States of America; 4 African Field Epidemiology Network, Dar es Salaam, Tanzania; 5 Ministry of Health Community Development Gender Elderly and Children, Government of Tanzania, Dar es Salaam, Tanzania; University of Ghana College of Health Sciences, GHANA

## Abstract

**Introduction:**

Effective point-of-care testing (POCT) is reliant on optimal specimen collection, quality assured testing, and expedited return of results. Many of the POCT are designed to be used with fingerstick capillary blood to simplify the blood collection burden. However, fingerstick blood collection has inherent errors in sampling. An evaluation of the use of capillary and venous blood with CD4 POCT was conducted.

**Methods:**

Three different specimen collection methods were evaluated for compatibility using the Alere Pima CD4 assay at 5 HIV/AIDS healthcare sites in Dar es Salaam, Tanzania. At each site, whole blood specimens were collected from enrolled patients by venipuncture and fingerstick. Pima CD4 testing was performed at site of collection on venipuncture specimens (Venous) and fingerstick blood directly applied to a Pima CD4 cartridge (Capillary-Direct) and collected into an EDTA microtube (Capillary-Microtube). Venous blood was also tested at the laboratory by the reference CD4 method and Pima for comparison analysis.

**Results:**

All three specimen collection methods were successfully collected by healthcare workers for use with the Pima CD4 assay. When compared to the reference CD4 method, Pima CD4 testing with the Capillary-Microtube method performed similarly to Venous, while Pima CD4 counts with the Capillary-Direct method were slightly more biased (-20 cells/μL) and variable (-229 to +189 cells/μL limit of agreement). Even though all three collection methods had similar invalid Pima testing rates (10.5%, 9.8%, and 8.3% for Capillary-Direct, Capillary-Microtube, and Venous respectively), the ability to perform repeat testing with Capillary-Microtube and Venous specimens increased the likelihood of acquiring a valid CD4 result with the Pima assay.

**Conclusions:**

Capillary blood, either directly applied to Pima CD4 cartridges or collected in an EDTA microtube, and venous blood are suitable specimens for Pima CD4 testing. The advantages of capillary blood collection in an EDTA microtube are that it uses fingerstick collection which mimics venous blood and allows extra testing without additional blood collection.

## Introduction

WHO currently recommends antiretroviral therapy (ART) for all HIV positive patient regardless of CD4 counts, but in many resource limited settings, CD4 counts are used in the management of HIV-positive patients to evaluate immune status and to direct treatments of suspected opportunistic infection [1,2]. CD4 testing is traditionally performed in a clinical laboratory and depending on the laboratory capacity, the results are typically available within 2 to 14 days after the HIV patient provides a specimen[3]. Problems exist in the management of HIV patient care when CD4 results are not utilized for reasons including, patient not providing a specimen or not returning to obtain their CD4 results[4]. To address this problem, healthcare programs are using point-of-care tests (POCT) to provide CD4 results when patients are at the site, which allows for better retention into care and reduces lost-to-follow up[5]. One such CD4 POCT is the Alere Pima CD4 assay[6].

Whole blood specimens collected by venipuncture have been widely used as the acceptable sample for CD4 testing. Although effective, venipuncture requires skilled phlebotomists, may cause anxiety in patients, and has biosafety concerns[7]. These venipuncture issues are relevant in high HIV/AIDS disease burden settings where the number of trained and skilled healthcare workers are scarce[8]. Capillary whole blood collected by fingerstick has been used as an alternative specimen collection method in children and with patients in which venipuncture is difficult and is an acceptable specimen form many POCT, incliding HIV rapid testing[7, 9]. The Pima CD4 assay is capable of using both venous and capillary (fingerstick) whole blood specimens.

For POCT, capillary whole blood specimens may have some inherent differences from venous specimens which could affect the accuracy and reliability. The drop-to-drop variation from capillary blood suggest caution when using measurement from a single drop of fingerstick blood[10]. This drop-to-drop variation may be eliminated by collecting a more homogenous specimen into a microtube blood collection device. Also, capillary blood specimens tend to have higher white and red blood cell counts, hematocrit, and hemoglobin levels and to be less precise due to variability in collection[11–14]. However, elevated white counts in capillary blood were mainly due to an increase in granulocyte cells with little effect from the lymphocyte population[14]. Furthermore, absolute CD4 count and percentages obtained using capillary whole blood have been shown to be in agreement with those obtained from venous blood [15, 16]. But, a number of studies have reported challenges with using fingerstick capillary blood in CD4 POCT, demonstrating the need for improvement in specimen collection methods and use in in CD4 POCT[17, 18]. Additionally, fingerstick collection for quantitative assays like CD4 testing requires techniques to establish a good blood flow, prevent hemolysis, and reduce the addition of interstitial fluids to the specimen[7, 9, 15].

We evaluated using fingerstick and venipuncture blood specimens with the Pima CD assay at HIV/AIDS care and treatment clinics in Dar es Salaam, Tanzania. We were able to assess the ability of the healthcare worker at the site to collect specimens and to generate accurate and reliable point-of-care CD4 results with the Pima CD4 assay. Included in this study was the addition of an EDTA microtube specimen collected by fingerstick and it comparison to fingerstick specimens directly applied to POCT device.

## Methods

### Study design

Informed consent was obtained from all patient and HCW participants in accordance with the United States Department of Health and Human Services’ 45 CFR 46 Protection of Human Subjects. Prior to study initiation, ethics approval was obtained from the Tanzania National Institute for Medical Research, Tanzanian Ministry of Health and Social Welfare. An evaluation of specimen types (capillary versus venous blood), and collection methods (direct application versus blood tube) was performed with the Pima CD4 assay. At the point-of-care site, healthcare workers (HCW) collected whole blood specimens for Pima CD4 testing from enrolled participants via fingerstick and venipuncture. Fingerstick blood was directly applied to the Pima CD4 cartridge and collected into an EDTA microtube. Pima CD4 testing was performed on each patient at the site of collection by the HCW on all 3 specimen collection types: fingerstick direct application (Capillary-Direct), fingerstick EDTA microtube (Capillary-Microtube), and venous EDTA whole blood (Venous). After Pima CD4 testing at the site, the each patient’s venous EDTA tube was transported to the reference laboratory for CD4 testing using a reference CD4 method and the Pima CD4 assay.

Five high-volume healthcare site in close proximity to the reference laboratory National Health Laboratory Quality Assurance Training Center that provided HIV care and treatment (CT) and Prevention of Mother to Child Transmission (PMTCT) were chosen for specimen collection and Pima testing. All sites were in Dar es Salaam. HIV-positive male and female patients between 8 and 65 years of age attending the selected sites and requiring a routine CD4 test were approached to participate in the study.

### Specimen collection

Fingerstick capillary blood collection was done by a standard method for a spontaneous blood flow with minimal hemolysis and blood dilution with interstitial fluid, S1 and S2 Figs [7, 9]. A single-used 1.6 x1.5 mm blade lancet (Safety-Lancet, Sarstedt, Inc) was used to prick the side of the third or fourth finger. After wiping away the first drop of blood, whole blood was collected into both the Pima CD4 cartridge and EDTA microtube (BD Microtainer, BD Diagnostic, USA). If necessary to increase the blood flow, slight pressure was applied to the finger above and distal to the puncture site. HCW were trained at the site on fingerstick collection by study trainer who is experienced and certified in phlebotomy. All HCW had to demonstrate competency in fingerstick collection prior to study start. Collection of peripheral blood via venipuncture was done using EDTA vacuum tubes (BD Vacutainer, BD Diagnostics, USA).

### CD4 count testing

The Pima CD4 assay (Alere Inc., Waltham, Maryland, USA) consists a of a Pima Analyzer, a portable bench-top fixed volume cytometer, used for processing and analysis of Pima CD4 cartridges to provide absolute CD4 counts (cells /μL). Nineteen Pima Analyzers were used to perform Pima CD4 testing at all five healthcare sites and at the NHLQATC. For Pima CD4 testing with Capillary-Microtube and Venous specimens, a blood sample was applied a Pima CD4 cartridge using a disposable transfer pipette. Each Pima CD4 cartridge was analyzed with the Pima Analyzer within one minute of applying blood to the cartridge. All healthcare workers and laboratorians performing Pima CD4 in this study were extensively trained on the Pima CD4 assay including use of and interpretation of quality control materials.

CD4 count testing using BD MultiTest CD3/CD4/CD8/CD45 reagent and Trucount tubes with BD FACSCalibur (Becton Dickinson, San Jose, California, USA) was performed at the NHLQATC with Venous specimens. BD MultiSet software was used to analyze results.

### Data analysis

All patient demographics and CD4 results were recorded in a Microsoft database and analyzed with Microsoft Excel. Pima CD4 data were exported directly from each Pima Analyzer and used to determine the number of Pima tests performed, invalid test rates, and types of invalid tests. The estimate of error was evaluated by scatter-plot and best-line analysis with linear regression to determine coefficient of determination (R^2^), slopes, and y-intercepts[19]. Bland-Altman analysis was done to determine systematic bias and imprecision of the Pima CD4 assay[20].

## Results

### Demographics and specimen collection

A total of 1060 patients from five HIV/AIDS healthcare sites were enrolled during a 5 week period in 2011. Four of the five healthcare sites enrolled between 164 to 198 patients and the other site enrolled 345 patients during this time period (Table 1). Of the total patients, 856 (81%) were female of which 162 (15% of total) were pregnant and receiving PMTCT service. A majority, 648 (61%) of the patients were on ARV treatment. The age range of the patients ranged from 8 to 65 years old, with 4% of the patients less than 18 years old and 7% greater than 49 years old.

**Table 1:** Demographic of patients enrolled by healthcare site.

						Age (years)				
	N =	Female	Male	On ARV	Pregnant	<18	18–49	>49	HCW^a^	Pima^b^
**Site 1**	176	143	30	115	15	13	154	8	3	2
**Site 2**	164	152	12	49	46	1	152	4	4	2
**Site 3**	345	241	104	262	10	25	275	42	2	5
**Site 4**	198	161	36	105	61	5	170	16	2	3
**Site 5**	177	159	18	117	30	3	163	8	2	3
**Reference Lab**									2	4
**Totals**	1060	85 (81%)	200(19%)	648(61%)	162(15%)	47(4%)	914(86%)	78(7%)	15	19

^a^ HCW: Number of Healthcare Worker/Laboratorian at each site performing Pima CD4 testing

^b^ Number of Pima Analyzers at each site

Overall, there were few compromised specimens due to difficulties or errors associated with blood collection. Of the 1060 patients, 25 (2.8%) of the fingerstick blood collections were of insufficient volume, 6 (0.6%) clotted, and 2 (0.2%) were reported as being difficult to collect (data not shown). Each of the compromised specimens resulted in the inability to acquire a CD4 result. Patients were allowed to refuse specimen collection at any time during the study and one patient did refuse fingerstick blood collections, while none refused venipuncture.

### Pima CD4 testing numbers and invalid test rates

A total of 15 HCW performed Pima CD4 testing on 19 Pima Analyzers (Table 1). Overall, there were 4536 Pima CD4 tests performed on whole blood specimens at the five healthcare sites and the NHLQATC reference laboratory (Table 2). The 4536 Pima CD4 test included tested of all 1060 patient specimen types at the healthcare sites and venous blood testing at the NHLQATC plus any repeat testing. Of the 4536 Pima tests, 469 (10.3%) were invalid with an error message reported by the Pima Analyzer. At the five healthcare sites, 316 (9.5%) of the 3317 Pima tests were invalid compared to 153 (12.6%) of the 1219 Pima tests at the NHLQATC laboratory. Fingerstick specimens directly applied to the Pima CD4 cartridge (Capillary-Direct) accounted for 1056 of the Pima tests at healthcare sites and included 111 (10.5%) invalid tests. Also at the healthcare sites, Capillary-Microtube and Venous specimens accounted for 1118 and 1143 of the Pima tests respectively, and included 110 (9.8%) and 95 (8.3%) invalid tests, respectively.

**Table 2:** Invalid Pima CD4 tests per testing site and specimen type.

		Healthcare Sites					
Pima Error Message	Possible Reasons	Capillary-Direct	Capillary-Microtube	Venous	Total	Lab Venous	Total
**Invalid Test Error 810**	**Channel filling**	30	7	7	44	5	49
**Invalid Test Error 820**	**Focus control**	0	0	1	1	0	1
**Invalid Test Error 830**	**Specimen integrity**	2	0	0	2	0	2
**Invalid Test Error 840**	**Dirt on cartridge**	1	1	1	3	0	3
**Invalid Test Error 850**	**Sample/bubbles**	42	38	27	107	57	164
**Invalid Test Error 860**	**Reagent/bubbles**	17	27	16	60	33	93
**Invalid Test Error 880**	**Cell movement/bubbles**	4	24	29	57	18	75
**Invalid Test Error 910**	**Image/bubbles/Dirt**	9	9	7	25	14	39
**Invalid Test Error 930**	**Homogeneity/unlikely**	1	1	0	2	1	3
**Invalid Test Error 940**	**Sample/dirt**	5	2	3	10	14	24
**Test not finished Error 200**	**Aborted test**	0	1	4	5	11	16
	**Total # Invalid Tests **	111	110	95	316	153	469
	**Total # Pima Tests **	1056	1118	1143	3317	1219	4536
	**% Invalid Tests**	10.5%	9.8%	8.3%	9.5%	12.6%	10.3%

The Pima Analyzer reports the error types for each of the invalid tests. We compiled these error messages to determine if a particular specimen type was prone to a specific invalid test(s) code (Table 2). Capillary-Direct specimens had the highest percentage (68% 30/44) of the channel filling error code, Invalid Test Error 810. At the healthcare sites, a majority (93% 53/57) of the Invalid Test Error 880 codes were associated with Capillary-Microtube and Venous specimens, which were possibly due to cell movement or bubbles in the Pima CD4 cartridge during testing. Invalid Test Error 850 code was 35% (164/469) of the total invalid Pima CD4 tests. This error code is likely to occur with improper loading and filling with blood into the Pima CD4 cartridge, resulting in errors that affect the Pima CD4 assay. However, Pima CD4 cartridge or Analyzer could not be totally ruled out as the cause of Invalid Test Error 850. Operator aborting the Pima testing (Error 200), either due to technical or mechanical problems with the Pima Analyzer or testing procedural errors caught by the operators, occurred in 3.4% (16/469) of all the invalid tests.

We analyzed the invalid Pima tests rates for each site to determine if a particular site was more prone to Pima testing errors per blood specimen type (Table 3). Site #1 had the lowest invalid test rate of 6.8% (37/544 Pima tests) compared to Site #4 with the highest 13.9% (91/653). The other three healthcare sites had between 8.3% and 9.9% invalid Pima test rates. At each site, the invalid Pima test rates were consistent between each of the specimen types, except at Site # 2 where 14.4% (25/174) of the Capillary-Direct specimens had a Pima invalid test compared to 6.9% (12/174) and 8.4% (15/179) of the Capillary-Microtube and Venous specimens, respectively.

**Table 3:** Healthcare sites invalid tests per specimen type.

		Capillary-Direct	Capillary-Microtube	Venous	Total
**Site #1**	**Total # Invalid Test **	13	11	13	37
	**Total # Pima Test **	174	182	188	544
	**% Invalid Test**	7.5%	6.0%	6.9%	6.8%
**Site #2**	**Total # Invalid Test **	25	12	15	52
	**Total # Pima Test **	174	174	179	527
	**% Invalid Test**	14.4%	6.9%	8.4%	9.9%
**Site #3**	**Total # Invalid Test **	26	35	26	87
	**Total # Pima Test **	332	361	355	1048
	**% Invalid Test**	7.8%	9.7%	7.3%	8.3%
**Site #4**	**Total # Invalid Test **	30	34	27	91
	**Total # Pima Test **	201	225	227	653
	**% Invalid Test**	14.9%	15.1%	11.9%	13.9%
**Site #5**	**Total # Invalid Test **	17	18	14	49
	**Total # Pima Test **	175	176	194	545
	**% Invalid Test**	9.7%	10.2%	7.2%	9.0%

### Pima CD4 results per specimen

HCW successfully collected whole blood by both fingerstick and venipuncture and performed POCT with the Pima CD4 assay. However with Capillary-Direct specimens, a Pima CD4 result could be acquired and recorded for only 90.1% (955/1060) of the patients (Table 4). This ability to acquire and record a CD4 result with Capillary-Direct specimens varied among the healthcare sites, ranging between 84.8 to 92.5%. Pima CD4 results were recorded from 95.5% (1012/106) of the Capillary-Microtube specimens, and varied between 94.9 to 98.2% at four of the healthcare sites. Site #5 recorded Pima CD4 results for only 88.7% of the Capillary-Microtube specimens. The ability to acquire CD4 results using the Pima CD4 assay was best with venipuncture specimens. Pima CD4 results were acquired for 99.1% (1050/1060) and 99.8% (1058/1060) of the Venous specimens at the healthcare sites and NHLQATC, respectively. Also at the NHLQATC, 99.5% (1055/1060) of the Venous specimens had CD4 results recorded with the reference method, Multiset reagent with FACSCalibur.

**Table 4:** Specimens type with recorded a Pima CD4 result per site.

		Pima CD4 Result Recorded		
	N = ^a^	Capillary-Direct	Capillary-Microtube	Venous
**Site 1**	176	161 (91.4%)	169 (96.0%)	174 (98.8%)
**Site 2**	164	149 (90.9%)	161 (98.2%)	164 (100%)
**Site 3**	345	319 (92.5%)	338 (98.0%)	342 (99.1%)
**Site 4**	198	168 (84.8%)	187 (94.4%)	194 (98.0%)
**Site 5**	177	158 (89.3%)	157 (88.7%)	176 (99.4%)
**Total**	1060	955 (90.1%)	1012 (95.5%)	1050 (99.1%)

^a^ Number of patients enrolled per site

Even though the invalid Pima test rate was similar for all three specimens at the healthcare sites, 10.5%, 9.8% and 8.3% for Capillary-Direct, Capillary-Microtube, and Venous, respectively; the ability to record a Pima CD4 result was different between the specimens, 90.1%, 95.5%, and 99.1% for Capillary-Direct, Capillary-Microtube, and Venous, respectively (Tables 2 and 4). This difference was due to the ability to perform repeat testing with Capillary-Mircotube and Venous specimens. During this study, the HCW were instructed to only perform one fingerstick per patient, although in a few situations a repeat fingerstick was performed by the HCW to acquire a Pima CD4 results.

The failure to acquire a CD4 result with Capillary-Direct specimens was mostly due to an invalid Pima test. With Capillary-Direct specimens, an invalid Pima tests contributed to 94 out of 105 CD4 results not recorded (Table 5). With no opportunity to do repeat testing with Capillary-Direct specimens, the event of an invalid Pima CD4 test resulted in a higher percentage of Capillary-Direct specimen failures to acquire a CD4 results. Similarly, although few in numbers, the majority times (90%, 9/10) for not being able to record a CD4 result from Venous specimens was due to invalid Pima tests.

**Table 5:** Reason for CD4 results recording failure by specimen type.

	Capillary-Direct	Capillary-MicroTube	Venous
**Pima Testing Error**	94	14	9
**Difficult Collection**	2	0	0
**Insufficient Volume**	5	25	0
**Blood Clotted**	0	6	0
**Not Tested**	3	2	1
**Patient Refused**	1	1	0
**Total**	**105**	**48**	**10**

### Pima CD4 accuracy with fingerstick and venipuncture specimens

**Fig 1:**
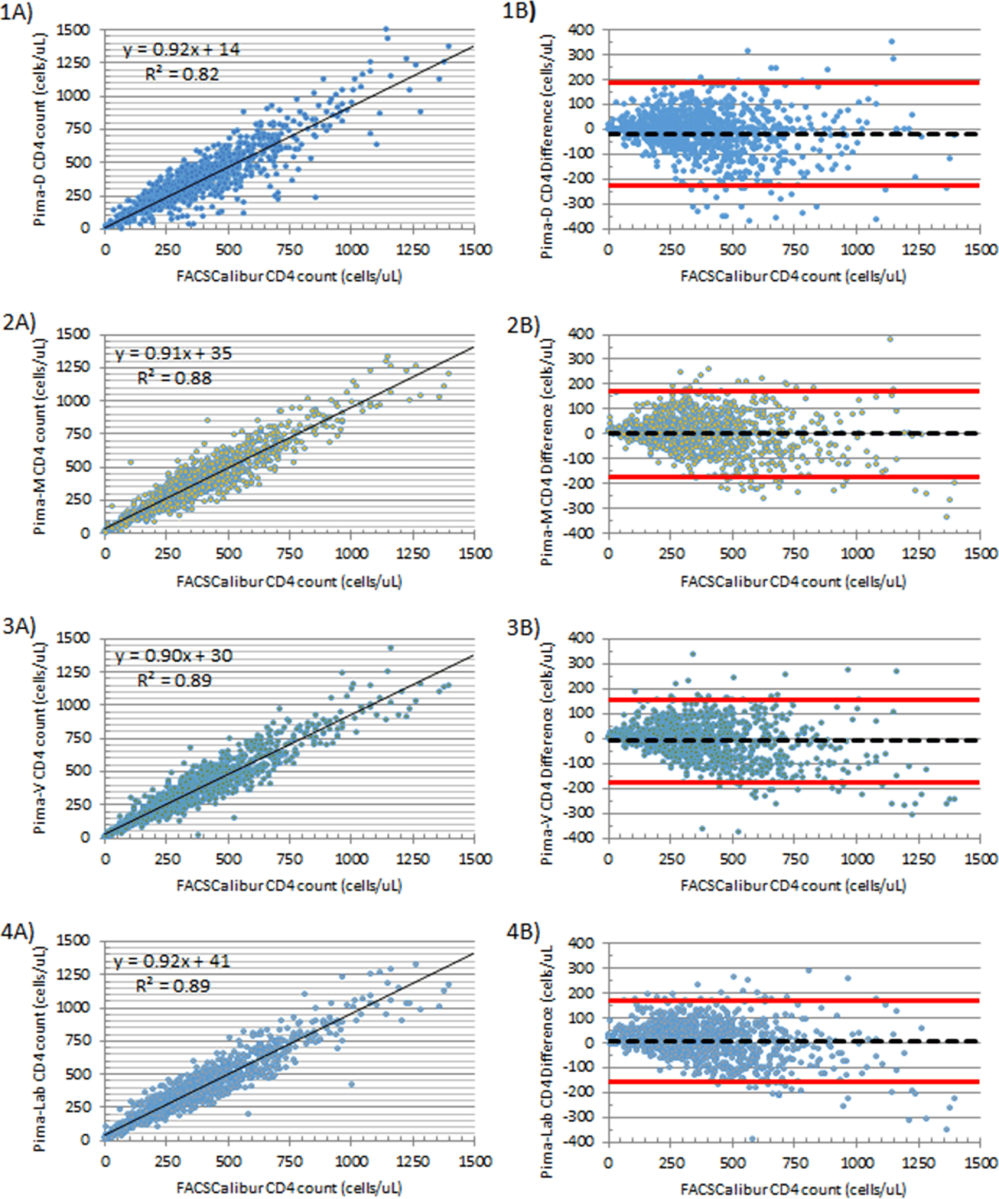
Scatter plots and Bland-Altman plots comparing the reference CD4 counts versus Pima CD4. Scatter and Bland-Altman plots for capillary blood directly applied to CD4 cartridges, Pima-D (1A and 1B), capillary blood collected in EDTA microtube, Pima-M (2A and 2B), venous blood,Pima-V (3A and 3B), or venous blood tested with the Pima at the reference laboratory, Pima-Lab (4A and 4B) with reference CD4 assay being BD Multitest reagent and BD Trucount Tubes using a BD FACSCalibur.

Pima CD4 testing with fingerstick and venipuncture whole blood specimens yielded absolute CD4 counts that were in close agreement with paired venous specimens tested with the reference method using Multiset reagent and Trucount on FACSCalibur (Fig 1 and Table 6). With fingerstick blood specimens directly applied to the Pima CD4 cartridge, scatter plot analysis estimated the best-fit line to have a slope of 0.92 and y-intercept of +14 with a R^2^ value of 0.82. Bland-Altman analysis with the same specimens estimates a bias of -20 cells/μL with a wide limit of agreement of -229 to +189 cells/μL. The scatter plot and Bland-Altman analysis of both Capillary-Microtube and Venous specimens tested at the healthcare site yielded similar results, with estimated slopes of 0.91 and 0.90, y-intercepts of +35 and +30, R^2^ value of 0.88 and 0.89, bias of 0 and-10, and standard deviation of bias of 86 and 83, respectively. Scatter plot and Bland-Altman analysis of Venous specimens tested at the reference laboratory with the Pima CD4 assay estimated the best-fit line slope to be 0.93, y-intercept +41 cells/ μL, y-intercept, R^2^ value 0.89, bias of +7 cell/μL, and limit of agreement from -157 to +170 cells/μL, similarly to those estimates generated with Capillary-Microtube and Venous specimens tested at the healthcare site.

**Table 6:** Summary of scatterplot and Bland-Altman analysis of Pima CD4 testing compared to reference CD4 method per location and specimen type.

		Linear Regression			Bland-Altman		
Pima Location	Specimen	Slope	Intercept	R^2^	Mean Bias	S.D.	Limit of Agreement
**Healthcare Site**	**Capillary-Direct**	0.92	+14	0.82	-20	104	-229, +189
**Healthcare Site**	**Capillary-Microtube**	0.91	+35	0.88	0	86	-171, +171
**Healthcare Site**	**Venous**	0.90	+30	0.89	-10	83	-175,+156
**Laboratory**	**Venous**	0.92	+41	0.89	+7	82	-157, +170

## Discussion

Collecting fingerstick blood in an EDTA microtube and or using venous blood has advantages over using a drop of fingerstick blood directly in a point-of-care testing cartridge. Data presented by Bond and Richards-Kortum showed higher average percent coefficient variation between drops of fingerpick blood when analyzing for cell counts or hemoglobin levels[10]. They also showed that the drop-to-drip variation is reduced with cumulative volume, as would be see in using an EDTA microtube with fingerstick collection. Our data also showed EDTA microtube capillary performed similar to venous blood in regards to accuracy when compared to a reference method. These finding should be taken in consideration of specimen type used for other quantitative POCT, such as those for HIV viral load testing.

Another advantage of collecting fingerstick blood in an EDTA microtube and venous blood is the ability to repeat a test without collecting another whole blood specimen from the patient. The ability to repeat the Pima CD4 test in situations when an invalid test occurs allows for a better chance of acquiring a CD4 test result from a patient; this was demonstrated with both Capillary-Microtube and Venous specimens. Also when tested with the Pima CD4 assay, fingerstick EDTA microtube specimens performed similarly to venipuncture blood collected in an EDTA tube. Results from scatter-plot and Bland-Altman analysis revealed that fingerstick EDTA microtube and venipuncture specimens were nearly identical when tested with the Pima CD4 assay and compared to a reference CD4 method. Others have seen similar results when testing flow cytometry based CD4 assay (FACSCalibur and FACSCount) with fingerstick blood collected in an EDTA microtube versus venipuncture specimens[15, 16]. All of this suggests that capillary EDTA microtube collected specimens resembles venipuncture whole blood and that collecting higher volumes of capillary blood reduces the variability reported with use of a single drop of fingerstick blood.

In HIV/AIDS patient management, multiple laboratory tests are needed for diagnosis of HIV infection and in the care and treatment, including laboratory tests to monitor the health status and detect possible opportunistic infection. Fingerstick blood collected in an EDTA microtube allows for multiple testing and reflex testing without additional blood collection. HIV rapid test could be performed with the EDTA microtube specimen, if positive, a CD4 count could be performed on this specimen, and if indicated a Cryptococcus antigen or complete blood count.

Improving access to CD4 testing with point-of-care testing in high-burden, resource-limited settings has played a critical role in the scale up of ART for HIV/AIDS care and linkage to treatment[21]. In order to improve access to testing, high quality and low-cost CD4 assays must be both available and suitable for use in resource-limited settings[8, 22]. But, having a quality CD4 assay does not guarantee a quality CD4 result. Acquiring an accurate and reliable CD4 result with POCT requires that whole blood specimens to be collected correctly, accurately identified, of sufficient volume, clot free and of good integrity, and that the HCW correctly performs the CD4 point-of-care assay. Failure to obtain a quality specimen or properly perform the CD4 assay will result in the inability to acquire a quality CD4 results for HIV/AIDS care.

All CD4 testing of HIV patients begins with proper blood collection. To obtain quality specimens it is essential to standardize the collection procedure and technique. This standardization is particularly important for fingerstick collection for quantitative assays, since capillary blood may also contain interstitial fluids which will affect the blood’s composition[7]. Also, in order to avoid hemolysis during the fingerstick procedure, correctly applied gentle pressure is only permissible if needed. The emphasis on stringent attention to fingerstick blood collection technique is vital for the success of CD4 POCT[17, 23, 24]. In this study, there were few compromised fingerstick specimens due to collection issues, with only 0.6% of the capillary blood specimens being clotted and 2.8% of the EDTA microtube being of insufficient volume. Our study suggests that training of the HCW on fingerstick collection and having them demonstrate competency prior to start of this study was important to obtain quality specimens.

This study was conducted in five high volume urban facilities where phlebotomy is performed on upwards of 100 clients per day. The HCW participating in this study were already skilled phlebotomists who were already experienced at fingersticking for other point-of-care tests, such as HIV rapid test. These HCW needed additional training on the fingerstick technique to obtain a quality specimen for a quantitative test, such as the Pima CD4 assay. We believe because of their experience and the high volume of participant in this study, the HCW were able to master the fingerstick technique resulting in few compromised specimens. A limit to our study is the HCW may not be representative of potential users in rural, peripheral facilities where Pima Analyzer may more likely be placed. Implementing the Pima CD4 assay in these rural low volume facilities may require more extensive training and monitoring.

Overall, in this study the Pima CD4 assay performance was in close agreement to the standard reference method. Pima CD4 testing at the healthcare site and laboratory estimated the bias was minimal for the different specimens and settings and a slight proportional systemic error with the Pima CD4 assay[19, 25]. Others have reported similar estimates of proportional systemic error with slopes, between 0.90 and 0.95, when comparing Pima CD4 with a reference method[24, 26–30]. This indicates that the Pima CD4 assay will underestimate the CD4 count, particularly with HIV patients with higher CD4 counts.

The Pima CD4 is capable of exporting the CD4 results and error codes for each of the invalid tests. Monitoring the number and rate of invalid Pima CD4 test is an important quality assurance tool to detect potential problems with the Pima Analyzer, Pima CD4 cartridge, and the operator performing the test. The Pima invalid tests are mostly due to problems with specimen integrity, improper filling of blood in the cartridge, reagent failure, analyzer failure, or if abort by the operator. If any of these problems arise, it is important that CD4 results are not reported as the quality of the CD4 results will be compromised. We compiled these error messages to determine if a particular error was common for these invalid tests or specimen type (Table 3). We found that a high proportion of Capillary-Direct specimens had Pima errors due to channel filling of blood into the Pima CD4 cartridge, indicating inherent difficulties may occur more often with direct application of capillary blood to the cartridge. With Capillary-Microtube and Venous specimens, a majority of the Pima errors may have been caused by introducing bubbles into the Pima CD4 cartridge during specimen loading. All this indicates for in order to obtain an accurate and reliable Pima CD4 result, both a quality specimen must be collected and correct processing of the blood into the Pima cartridge is necessary.

In summary, use of the Pima CD4 assay in a point-of-care setting was acceptable for all specimen types and collection methods. With fingerstick capillary blood specimens, there was an advantage of collecting this blood in an EDTA microtube. This specimen closely resembles that of venous blood in the performance on the Pima CD4 assay and allows for repeat testing when indicated.

### Disclaimers

The findings and conclusions in this report are those of the authors and do not necessarily represent the views of the U.S. Centers for Disease Control and Prevention.

This research has been supported by the President’s Emergency Plan for AIDS Relief (PEPFAR) through the Centers for Disease Control and Prevention.

Use of trade names is for identification purposes only and does not constitute endorsement by the U.S. Centers for Disease Control.

## Supporting information

S1 Fig Pima fingerstick blood collection. A job aid detailing fingerstick blood collection with direct application to a Pima CD4 cartridge.
(TIF)

S2 Fig EDTA Microtube fingerstick blood collection. A job aid detailing fingerstick blood collection into an EDTA microtube.
(TIF)

S1 File CD4 counts database. (XLSX)
